# Renal Microsporidiosis in Pediatric Bone Marrow Transplant Recipients: A Case Series

**DOI:** 10.5146/tjpath.2017.01416

**Published:** 2020-01-15

**Authors:** Saloni Shah, Sheba Sweetline Jacob, Rama Mani, Ashok Parameswaran, Sunil Kumar, Rajeev A Annigeri, Raja Mahesh, Ramya Uppuluri

**Affiliations:** Department of Histopathology, Apollo Hospitals, Chennai, Tamil Nadu, India; Department of Nephrology, Apollo Hospitals, Chennai, Tamil Nadu, India; Department of Hematology, Apollo Hospitals, Chennai, Tamil Nadu, India

**Keywords:** Microsporidiosis, Opportunistic infection, Pediatrics, Bone marrow transplantation

## Abstract

Microsporidiosis is a rare, but emerging opportunistic infection in solid organ transplant and stem cell transplant recipients. Renal involvement in microsporidiosis is very rarely seen in these recipients. We describe two cases of pediatric renal microsporidiosis, diagnosed on renal biopsies, following bone marrow transplantation presenting as severe acute kidney injury. The first patient died, whereas the second survived due to early diagnosis based on high index of suspicion and prompt treatment with Albendazole. We believe these are the first such reported cases of renal microsporidiosis in pediatric bone marrow transplant recipients.

## INTRODUCTION

Microsporidia are obligate, ubiquitous, intracellular parasites resembling fungi, which infect both vertebrates and invertebrates. More than 1200 species belonging to 143 genera of these parasites have been identified till date, of which 14 species are known to infect humans (1). These opportunistic protozoans are encountered usually in HIV infected and only occasionally in solid organ and bone marrow transplant recipients. Many intestinal and few extra intestinal microsporidial infections have been reported in solid organ and bone marrow transplant recipients. Renal allograft microsporidiosis in renal transplant patients (2), pulmonary (3) and disseminated forms in bone marrow transplant recipients, and the ocular form (4) in a corneal graft recipient, though very rare, are being reported in increasing frequency since early 1900s.

Human microsporidiosis is an important newly emerging opportunistic disease occurring in HIV-infected individu-als, severely immunocompromised patients such as solid organ (SOT) and stem cell transplant (SCT) recipients, travellers to tropical countries, the elderly, and children with malignancies (5). The depressed cell mediated immunity in these patients predisposes them to the infection. Microsporidia are transmitted by direct contact, through broken skin or eye membrane, trauma, by sexual intercourse in humans, and by vertical transmission in animals (6). However, it is still unclear if the infection is transmitted through the donor graft or due to predisposition following immunosuppression itself in the SOT and SCT recipients.

We report renal microsporidiosis causing acute kidney injury (AKI) in two pediatric bone marrow transplant (BMT) recipients, which to the best of our knowledge are the first such documented cases in this population.

## CASE REPORTS

### Case 1

**Figure 1:**
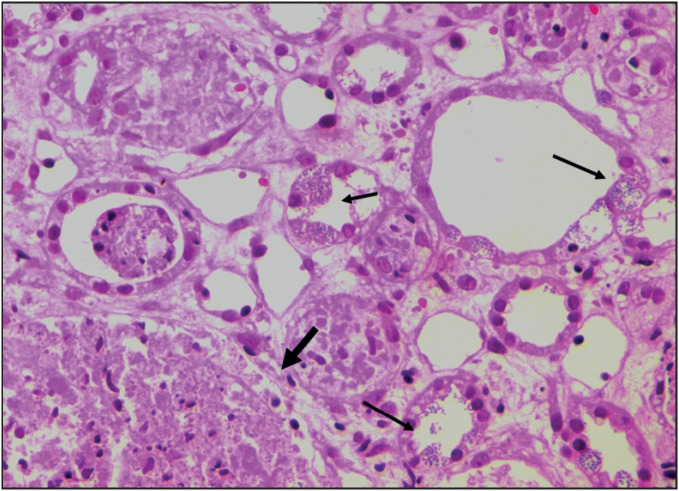
Renal biopsy with global glomerular necrosis (thick arrow), luminal and intracytoplasmic microsporidial organisms within the tubules (thin arrows) (H&E; x100).

**Figure 2:**
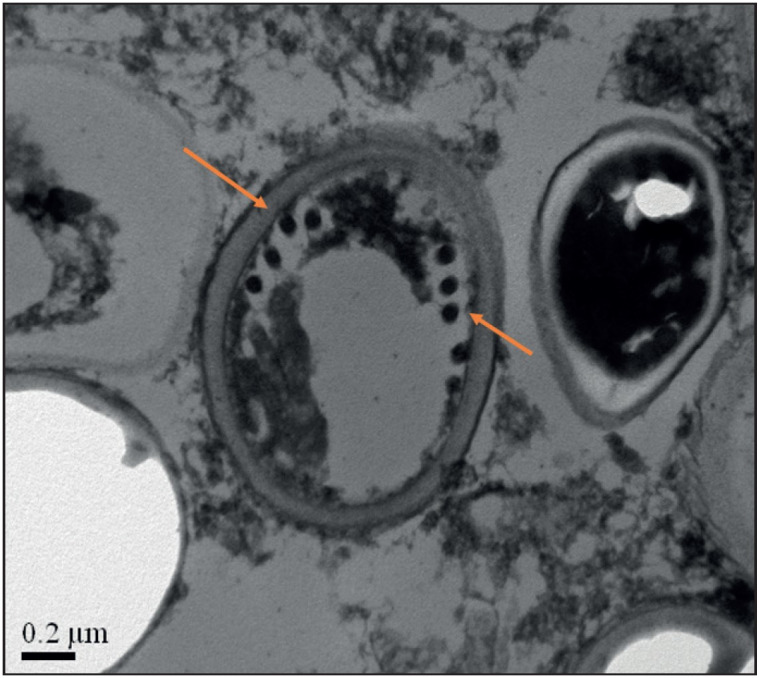
Electron micrograph demonstrating oval spores with single row of multiple polar tubes consistent with Encephalitozoons (Toluidine blue; x40000).

A one-and-a-half-year-old boy presented to our centre in September 2014 with fever, hepatosplenomegaly, bicytopenia and intracerebral haemorrhage. Bone marrow aspiration and biopsy were consistent with acute lymphoblastic leukemia (ALL). He received induction chemotherapy that included vincristine, daunorubicin, dexamethasone and intrathecal methotrexate. Post induction, he developed knee joint pain and fever. X rays revealed lytic lesions of the pelvic bone and femur. The aspiration and biopsy of the lytic bone lesions revealed myeloblasts. In view of this revised diagnosis of biphenotypic leukemia, high dose chemotherapy consisting of fludarabine, cytarabine, idarubicin and melphalan was given and haplo-identical hematopoeitic stem cell transplantation was performed on 23rd September 2014. On 104th day after transplantation, he presented with fever, abdominal distension, anasarca and breathlessness. He developed non oliguric AKI and azotemia increased progressively. He was initiated on peritoneal dialysis when the blood urea increased to 197 mg/dl. Serial ultrasonography of the abdomen showed progressive bilateral nephromegaly. In view of severe AKI, a percutaneous renal biopsy was done to rule out leukemic infiltration. The biopsy was received in 10% neutral buffered formalin on which histopathological examination and subsequently transmission electron microscopy were performed. On hematoxylin and eosin stained sections, the renal tissue showed diffuse and global glomerular necrosis admixed with histiocytes, mononuclear cells and degenerated cells. (Figure 1). The tubules showed diffuse simplification with intracytoplas-mic and luminal microsporidial organ-isms on Giemsa stain. Transmission electron microscopy confirmed the presence of microsporidia, with multiple oval and distorted spores displaying a single row of multiple polar tubes and ill-defined nuclei consistent with Encephalitozoons (Figure 2). Albendazole was started to treat the parasitic infection along with intravenous antibiotics to treat the lung consolidation. He continued to have fever spikes, leukopenia, thrombocytopenia and developed progressing respiratory distress. He subsequently developed encephalopathy, possibly secondary to sepsis. His condition worsened progressively and he expired.

### Case 2

**Figure 3:**
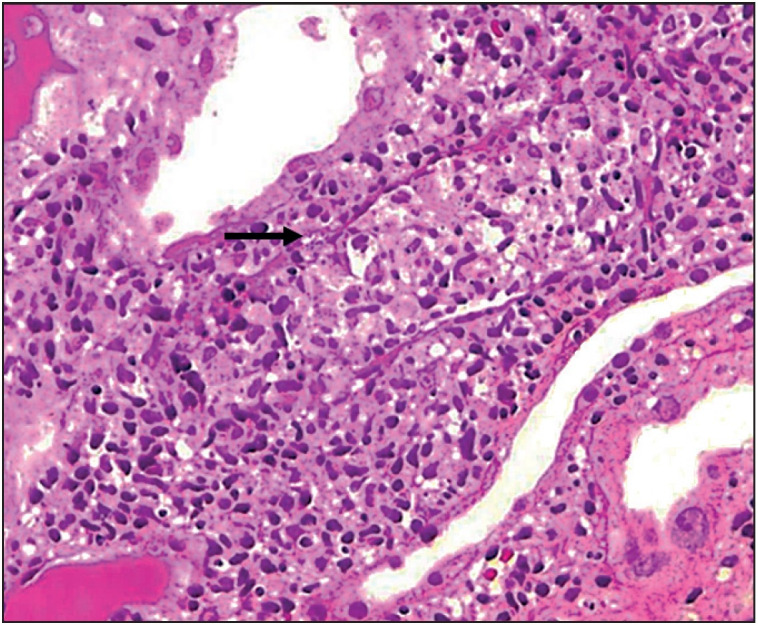
Interstitial granulomatous inflammation (PAS; x400).

**Figure 4:**
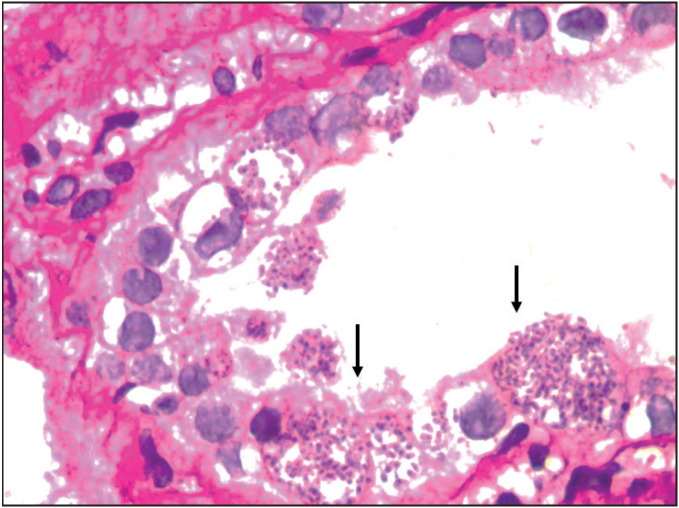
Intraepithelial and luminal microsporidia in the tubules (PAS; x400).

**Figure 5:**
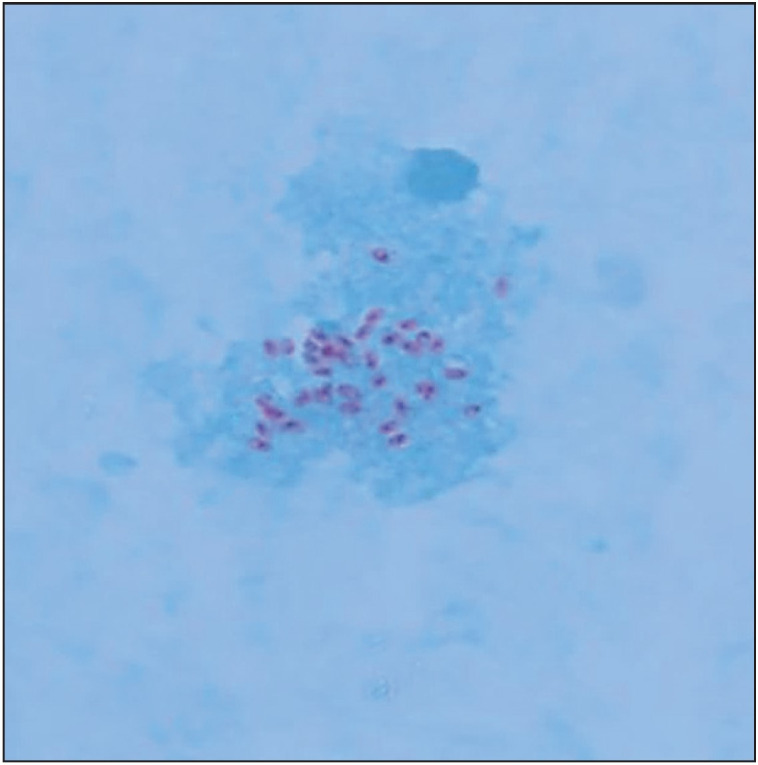
Urine deposit showing the red spikes of the microsporidial spores (Trichrome; x400).

A seventeen-year-old girl presented our hospital with fever, joint pain, intracerebral petechiae and haemorrhage on November 2014. Investigations revealed hyperleukocytosis and a lymph node biopsy was diagnostic of cortical T cell leukemia. She was treated with ablative chemotherapy followed by allogenic BMT in February 2015. On day 110 after BMT, she developed fever, lethargy, and prerenal azotemia along with drowsiness and altered behaviour. She was started on antibiotics and other supportive medications. However, she developed azotemia and a percutaneous renal biopsy was performed to determine the cause of azotemia. Histopathological examination showed unremarkable glomeruli. The interstitium showed granulomatous inflam-mation along with mononuclear infiltrates (Figure 3). The tubules showed luminal and intraepithelial PAS positive organisms consistent with microsporidia (Figure 4). Urine microscopic examination showed microsporidial spores on Trichrome stain (Figure 5). The patient was given albendazole for seven days. The fever subsided but she had worsening of AKI requiring dialysis for ten days. Subsequently, renal function improved gradually and returned to normal eventually.

## DISCUSSION

Microsporidiosis as an opportunistic pathogen came into the limelight with the emergence of the HIV epidemic. However, microsporidiosis is quite rare in non-HIV populations. Less than 100 cases of microsporidiosis occurring in HIV-seronegative SOT and SCT recipients have been reported worldwide since 1993. In SOT recipients, microsporidiosis presents more commonly as an intestinal infection or occasionally in disseminated form, but very rarely affects the kidneys in isolation. Pulmonary involvement is the commonest presentation in SCT recipients, followed by the disseminated form. However, no case of isolated renal microsporidiosis has been reported as yet in the latter group.

The commonly reported microsporidial pathogens affecting transplant recipients belong to the Enterocytozoon and Encephalitozoon species (7). These are characterised ultrastructurally by resistant spores with a coiled polar filament that inject the spore cytoplasm into the host cell under appropriate conditions (8). These spores are extruded out in the stool, urine, and sputum and can also be demonstrated in many tissues potentially presenting as nephritis, pneumonitis, keratoconjunctivitis, myositis, laryngitis and encephalopathy.

Several prospective and retrospective studies across the globe have reported microsporidial infection in transplant recipients. Liguory et al. from France studied microsporidial infection in stool specimens of 100 patients over a period of six years, of which 8 were organ transplant recipients (9). Another French study by Rabodoniria et al. reported 23 cases of microsporidiosis in transplant recipients, of which 5 kidney transplant recipients were positive for microsporidiosis in a phylogenetic analysis on *E. bieneusi* isolates (10). Bednarska et al. conducted a study on medically induced immunosuppressed adults in which 8 of 48 patients (17%) had intestinal microsporidiosis (11). A recent Indian study by Ghosal and collaborators done over a period of 9 years reported 16 (5.8%) cases of intestinal microsporidiosis in 272 renal transplant recipients (12).

In the pediatric population, only four cases of microsporid-iosis have been reported in SOT recipients and none in SCT recipients. Two seronegative girls from Cape Town in 2012 received a renal transplant from the same deceased donor for end-stage renal disease (ESRD) of uncertain etiology and ESRD secondary to steroid resistant nephrotic syndrome respectively and presented with pyrexia, diarrhea and deteriorating renal function a few months after the transplant. Faecal smears were clean. Renal biopsy and urine examination revealed microsporidial spores. They were treated with Albendazole and had disease-free survival (2). Recently, Desoubeaux et al. reported two young sisters with double liver-kidney transplant who developed intestinal microsporidiosis for which Fumagillin treatment was administered successfully (13). In adults, 4 cases of pulmonary and disseminated microsporidiosis respectively following allogenic bone marrow stem cell transplantation have been documented (3,14).

Microsporidial nephritis is a rare manifestation in SOT and SCT recipients. Renal microsporidiosis can occur as part of disseminated disease. However, very few cases of isolated renal microsporidiosis have been reported in the literature (11,15–17). Patients with renal microsporidiosis present with longstanding fever, AKI and deteriorating graft function about three months after the commencement of immunosuppressive therapy and a similar pattern was observed in our patients. Renal microsporidiosis can be demonstrated by light microscopic examination of the tissue biopsy by Hematoxylin and eosin, Toluidine blue, Warthin Starry, and Brown and Brenn stains. Urine demonstrates red ovoid spores with a clear vacuole-like zone and a diagnostic stripe representing the polar filament on the Trichrome stain (7). Kidneys with microsporidial nephritis usually contain spores in the tubular epithelium along with tubular necrosis, granulomatous interstitial nephritis, and the glomeruli are typically spared (18). Transmission electron microscopy is essential to validate the diagnosis of microsporidial infection and also for its speciation. Microsporidia demonstrate a single row of 4-7 coiled polar tubes that occur in a parasitophorous vacuole and presence of posterior vesicles. *E. intestinalis* is differentiated from *E. hellum* and *E. cuniculi* by honeycombing of its parasitophorous vacuole (1,18). However, complementary studies using molecular methods and antigenic probes are required for accurate speciation. *E. intestinalis* and *E. cunilis* are the commonest microsporidia to affect the kidney.

Albendazole is effective in most microsporidial species and is the drug of choice to treat intestinal and disseminated microsporidiosis. However, Albendazole is less effective against *E. bieneusi* (1). Fumagillin is an alternative drug that is especially very effective against *E. bieneusi*. Reversible thrombocytopenia and reduction in tacrolimus levels can occur following treatment with Fumagillin and hence patients should be monitored for these complications while on this drug (18,19).

The diagnosis of microsporidiosis should be considered in a febrile SOT or SCT recipient when tests for routinely encountered pathogens are unrevealing. Renal involvement in transplant recipients should be suspected in a febrile patient with AKI when the cause is unclear. Evaluation of the urine sediment by microscopy using special stains and renal biopsy could identify renal microsporidial infection. Identification of the pathogen was late and disease was disseminated, resulting in a fatal outcome in our first case. Hence, early identification of the pathogen is critical, since antimicrobial therapy is effective and curative as demonstrated in our second case.

To the best of our knowledge, these are the first reported cases of renal microsporidiosis causing severe AKI in pediatric SCT recipients and the first such, from India.

In conclusion, renal microsporidiosis is an unusual infection with a high mortality rate if untreated, typically occurring in a setting of immunosuppression which presents with AKI. We present two cases of renal microsporidiosis in pediatric SCT recipients, the first such cases reported in this population, to create awareness amongst transplant physicians and pathologists. One of our patients survived, possibly due to a high index of suspicion, leading to prompt diagnosis and early initiation of treatment.

## References

[ref-1] Didier Elizabeth S., Weiss Louis M. (2006). Microsporidiosis: current status. Curr Opin Infect Dis.

[ref-2] Ladapo Taiwo A., Nourse Peter, Pillay Komala, Frean John, Birkhead Monica, Poonsamy Bhavani, Gajjar Priya (2014). Microsporidiosis in pediatric renal transplant patients in Cape Town, South Africa: two case reports. Pediatr Transplant.

[ref-3] Teachey D. T., Russo P., Orenstein J. M., Didier E. S., Bowers C., Bunin N. (2004). Pulmonary infection with microsporidia after allogeneic bone marrow transplantation. Bone Marrow Transplant.

[ref-4] Kakrania R., Joseph J., Vaddavalli P. K., Gangopadhyay N., Sharma S. (2006). Microsporidia keratoconjunctivitis in a corneal graft. Eye (Lond).

[ref-5] Galván A. L., Sánchez A. M. Martín, Valentín M. A. Pérez, Henriques-Gil N., Izquierdo F., Fenoy S., Aguila C. (2011). First cases of microsporidiosis in transplant recipients in Spain and review of the literature. J Clin Microbiol.

[ref-6] Didier Elizabeth S., Weiss Louis M. (2011). Microsporidiosis: not just in AIDS patients. Curr Opin Infect Dis.

[ref-7] Garcia Lynne S. (2002). Laboratory identification of the microsporidia. J Clin Microbiol.

[ref-8] Bouzahzah Boumediene, Weiss Louis M. (2010). Glycosylation of the major polar tube protein of Encephalitozoon cuniculi. Parasitol Res.

[ref-9] Liguory O., Sarfati C., Derouin F., Molina J. M. (2001). Evidence of different Enterocytozoon bieneusi genotypes in patients with and without human immunodeficiency virus infection. J Clin Microbiol.

[ref-10] Rabodonirina Meja, Cotte Laurent, Radenne Sylvie, Besada Emilio, Trepo Christian (2003). Microsporidiosis and transplantation: a retrospective study of 23 cases. J Eukaryot Microbiol.

[ref-11] Bednarska Małgorzata, Bajer Anna, Siński Edward, Wolska-Kuśnierz Beata, Samoliński Bolesław, Graczyk Thaddeus K. (2014). Occurrence of intestinal microsporidia in immunodeficient patients in Poland. Ann Agric Environ Med.

[ref-12] Ghoshal U., Khanduja S., Pant P., Prasad K. N., Dhole T. N., Sharma R. K., Ghoshal U. C. (2015). Intestinal microsporidiosis in renal transplant recipients: Prevalence, predictors of occurrence and genetic characterization. Indian J Med Microbiol.

[ref-13] Desoubeaux G., Maakaroun-Vermesse Z., Lier C., Bailly E., Morio F., Labarthe F., Bernard L., Chandenier J. (2013). Successful treatment with fumagillin of the first pediatric case of digestive microsporidiosis in a liver-kidney transplant. Transpl Infect Dis.

[ref-14] Ambrosioni J., Delden C., Krause K. H., Bouchuiguir-Wafa C., Nagy M., Passweg J., Chalandon Y. (2010). Invasive microsporidiosis in allogeneic haematopoietic SCT recipients. Bone Marrow Transplant.

[ref-15] Talabani Hana, Sarfati Claudine, Pillebout Evangeline, Gool Tom, Derouin Francis, Menotti Jean (2010). Disseminated infection with a new genovar of Encephalitozoon cuniculi in a renal transplant recipient. J Clin Microbiol.

[ref-16] Gamboa-Dominguez Armando, De Anda Jazmin, Donis Jose, Ruiz-Maza Francisco, Visvesvara Giovinda S., Diliz Hector (2003). Disseminated encephalitozoon cuniculi infection in a Mexican kidney transplant recipient. Transplantation.

[ref-17] Nagpal A., Pritt B. S., Lorenz E. C., Amer H., Nasr S. H., Cornell L. D., Iqbal S., Wilhelm M. P. (2013). Disseminated microsporidiosis in a renal transplant recipient: case report and review of the literature. Transpl Infect Dis.

[ref-18] Latib M. A., Pascoe M. D., Duffield M. S., Kahn D. (2001). Microsporidiosis in the graft of a renal transplant recipient. Transpl Int.

[ref-19] Champion L., Durrbach A., Lang P., Delahousse M., Chauvet C., Sarfati C., Glotz D., Molina J.-M. (2010). Fumagillin for treatment of intestinal microsporidiosis in renal transplant recipients. Am J Transplant.

